# Automatic control of arterial blood pressure: computer, benchtop, and animal models

**DOI:** 10.3389/fmedt.2026.1955568

**Published:** 2026-10-15

**Authors:** Raghav Sriram, Bijan Abar, Siri Gullapalli, Yuhe Hu, Akshar Patel, Joshua Seth Broder

**Affiliations:** 1 Department of Emergency Medicine, School of Medicine, Duke University, Durham, NC, United States; 2 Lampe Joint Department of Biomedical Engineering, NC State University, Raleigh, NC, United States; 3 Pratt School of Engineering, Duke University, Durham, NC, United States

**Keywords:** animal model, automatic, benchtop analysis, blood pressure control, closed-loop, computer model

## Abstract

**Background:**

Timely, accurate, and stable control of blood pressure (BP) in critical illness is essential for good clinical outcomes. The current clinical standard entails human operators intervening between the monitor and infusion pump, introducing delay, potential error, and risk of over- and undershoot of therapeutic goals. We developed and tested a closed-loop, automatic control system for BP using computer, benchtop, and animal models.

**Methods:**

All models employed a proportional-integral-derivative (PID) algorithm to control mean arterial pressure (MAP). The three components are summed to determine the final output, corresponding to the infusion rate of fluids or vasoactive drugs. The computer model simulated an automatically controlled drug infusion to regulate MAP. The benchtop model added physical components (clinical cardiac monitor, microcontroller, medication pump, and pressure transducer), adjusting the hydrostatic pressure in a closed system to meet a target. In the animal model, the MAP of anesthetized swine undergoing surgery was regulated using an automatically controlled norepinephrine or clevidipine infusion. For the animal model, statistical inference and comparison between trials could not be made given the sequential, non-independent design; quantities are descriptive only, not inferential. We applied common control system metrics including rise time, settling time, and on-target percentage (OTP).

**Results:**

The computer model rapidly reached and maintained target MAP ± 5 mmHg with OTP of 90% (95% CI 89.4%, 90.6%) at 240 simulated seconds (ss) and 95% (95% CI 94.6%, 95.4%) at 294 ss. The benchtop system reached and maintained target MAP with a mean rise time of 0.47 ± SD 0.15 min, mean settling time of 0.62 ± SD 0.18 min, and mean OTP (using a tolerance of MAP ± 5 mmHg) of 91.25 ± 9.68%. In a swine model, automatic norepinephrine augmented MAP by > 15 mmHg with mean rise time of 2.25 (SD 0.65) min and mean OTP of 56.9% (SD 22.7%). No trial maintained an equilibrium value of target MAP ± 5 mmHg; therefore, no settling time could be calculated. Clevidipine decreased MAP by >15 mmHg with mean rise time of 1.00 (SD 0.40) min, mean settling time of 2.19 (SD 1.45) min, and mean OTP of 93.8% (SD 7.18%). The results of the swine model were confounded by concurrent non-survival surgery. Within this limitation, the swine model demonstrated that the automatic control system was technically feasible.

**Conclusions:**

These proof-of-concept studies utilizing computer, benchtop, and animal models provide initial evidence that an automatic PID algorithm has potential to control BP across a range of starting BP. Future studies can optimize the system and compare its performance with human operators in advance of potential human clinical trials.

## Introduction

Timely, accurate, and stable control of blood pressure (BP) in critical illness is essential for good clinical outcomes; values and rates of change that are too high, too low, too fast, or too slow are all associated with negative outcomes (1). An unmet clinical need exists for systems to achieve these goals.

For general hypertensive emergencies, guidelines call for BP to be decreased by 20%–25% in the first hour, followed by a target of 160/100 mmHg between hours 2 and 6 (1, 2). Further complicating management, specific acute hypertensive emergencies (e.g., intracerebral hemorrhage, ischemic stroke, acute coronary syndromes, heart failure, and aortic dissection) each have unique guidelines for target BP and speed of BP reduction (Table 1) (1). Even in a closely monitored trial setting, a minority of hypertensive patients meet treatment goals for lowering BP; in the INTERACT2 trial of BP control in hemorrhagic stroke, only 33.4% of patients in the intensive treatment group met the target systolic BP < 140 mmHg at 1 h (3). Moreover, excessive BP reduction (e.g., hypotension requiring cessation of IV therapy) and excessively rapid BP reduction are also common and associated with adverse outcomes (4, 5). In the Studying the Treatment of Acute hyperTension (STAT) registry, hypotension requiring cessation of IV antihypertensive therapy occurred in 6% of hypertensive patients with neurological emergencies (e.g., subarachnoid hemorrhage, traumatic brain injury, and hemorrhagic and ischemic stroke) and was associated with 3.5-fold mortality (5). In the same registry, in patients with acute hypertensive heart failure, systolic BP below 120 mmHg within 12 h occurred in 18% of patients and was associated with 2.4 times risk of a composite outcome (mechanical ventilation, intensive care unit admission, hospital length of stay ≥7 days, and death at 30 days) (4).

**Table 1:** Blood pressure goals for selected hypertensive and hypotensive emergencies.

Condition	Immediate blood pressure goals	Delayed blood pressure goals/other recommendations
Malignant hypertension (1)	Decrease BP by 20%–25% in the first hour	Decrease BP to 160/100 mmHg by hours 2–6
Hypertensive encephalopathy (1)	Decrease BP by 20%–25% in the first hour	Decrease BP to 160/100 mmHg by hours 2–6
Acute intracerebral hemorrhage (1, 3)	Decrease systolic BP to 140–150 mmHg within 1 h	Avoid lowering systolic BP below 140 mmHg Maintain systolic BP > 180 mmHg if large hematoma volume or elevated intracranial pressure
Acute ischemic stroke (1)	If thrombolytic therapy is indicated, decrease BP to <185/110 mmHg before giving thrombolytic agents and maintain BP <180/105 mmHg for the first 24 h	If thrombolytic therapy is not indicated and there is no acute target organ damage other than stroke, the strategy depends on BP. If BP is <220/120 mmHg, no intervention is indicated for the first 48–72 h. If BP is ≥220/120 mmHg or there is other acute target-organ damage, such as heart failure or myocardial infarction, decrease BP by 15% within 1 h.
Acute coronary syndromes (1)	Decrease systolic BP to <140 mmHg within 1 h	Maintain diastolic BP >60 mmHg
Acute heart failure (1)	Decrease systolic BP to <140 mmHg within 1 h	—
Aortic dissection (1)	Decrease systolic BP to <120 mmHg within 20 min	Decrease heart rate to <60 beats/min within 20 min
Septic shock (8)	Initial MAP target of 65 mmHg (± 5 mmHg)	“In practice, it is not feasible to maintain MAP at exactly 65 mmHg, so a reasonable range (e.g., within 5 mmHg) should be used. Vasopressors should be titrated to maintain MAP within this range.” (8)
Postcardiac arrest or cardiogenic shock (9)	MAP ≥65 mmHg	“Hypotension should be avoided in adults after ROSC [Return Of Spontaneous Circulation] by maintaining a minimum MAP of at least 65 mmHg.” (9)
Traumatic hemorrhagic shock, prehospital (10)	Systolic BP 80–100 mmHg	Systolic BP >100 mmHg if traumatic brain injury is suspected

Adapted from the *New England Journal of Medicine*, *Society for Critical Care Medicine*, *Surviving Sepsis Campaign International Guidelines, American Heart Association, Western Trauma Association* (1, 8–10).

Even when target BP is initially achieved, volatility is common. In the STAT registry of 1,588 United States patients with acute severe hypertension requiring admission, 60% of patients experienced re-elevation after initial control, and 4% had iatrogenic hypotension requiring treatment (6). In a similar European registry of 791 patients (Euro-STAT), 13% of patients had recurrent severe hypertension after initial control, and intravenous antihypertensive therapy was associated with clinically significant hypotension in 10% of patients (7).

For hypotensive emergencies (shock), rapid normalization and maintenance of BP to sustain perfusion are essential, with guidelines generally recommending a minimum mean arterial pressure (MAP) of 65 mmHg (Table 1) (8, 9). Persistent hypotension is associated with increased mortality in septic and cardiogenic shock (11, 12). However, exceeding a minimum perfusion pressure may be detrimental to some shock states. A systematic review found no mortality benefit and an association with greater risk of supraventricular cardiac dysrhythmias for MAP targets >70 mmHg in critically ill, hypotensive patients treated with vasopressors (13). In traumatic hemorrhagic shock, higher BPs are hypothesized to disrupt hemostatic clot and lead to increased bleeding and mortality. A meta-analysis suggests a mortality benefit using permissive hypotension (systolic BP 50–70 mmHg or MAP ≥50 mmHg, compared with higher systolic BP 65–100 mmHg or MAP ≥65 mmHg) in adult trauma patients with hemorrhagic shock (14). The balance of bleeding risk and risk of hypoperfusion, particularly in patients with traumatic brain injury, remains an area of active research (15).

The current clinical standard for BP control entails human operators intervening between two digital devices (a monitor and infusion pump), introducing delay, potential for error, and risk of over- and undershoot of BP values and rates of change relative to therapeutic goals. Manual adjustment of vasopressor and vasodilator infusions has other substantial impacts on patient safety, provider time resources, and fatigue. Intensive care unit nurses following detailed instructions for norepinephrine infusion (titrating dose by 2–5 µg/min every 1–2 min) required a median of 32 min (IQR 12–65) to achieve hemodynamic goals (16). Maintaining target BP is challenging even with close attention in a research setting; a randomized controlled trial of vasopressors in shock found that 68%–69% of hourly MAP measurements were outside of the target range (17).

A better solution to the problem of BP control may be found in controls engineering, a field specializing in the use of closed-loop feedback to achieve specified targets. Examples include thermostatic control, autopilot systems for aircraft, and (in medicine) glucose monitoring and insulin delivery systems. Previous investigators have explored the use of control algorithms for autoregulation of BP, primarily in computer or single-modality settings, or in hypotensive resuscitation via fluid administration (18–22). We have developed a control system for BP, tested progressively across computer, benchtop, and live animal platforms, each with its own benefits and limitations (Table 2), and capable of fluid or vasoactive drug treatment of both hypo- and hypertension. We hypothesized that this control system can automatically modulate BP. We present findings from each of the platforms and discuss next steps for research.

**Table 2:** Comparison of features of computer, benchtop, animal, and human models using an automatic PID algorithm.

Model	Versatility	Speed	Logistical ease	Cost	Risk	Clinical realism
Computer	High^a^	High^b^	High	Low	None	Uncertain
Benchtop	Limited^c^	Moderate^d^	High	Low	None	Uncertain/low
Animal	Moderate^e^	Moderate^f^	Low	High	Low	High
Human	Low^g^	Low^h^	Low	High	High	Highest

a Can model different PID terms, medical conditions (sepsis, cardiogenic shock, hypovolemic shock, and hypertensive emergencies), different medications, and patient physiology/responsiveness to medications. Safety/features can be modeled, e.g., maximum infusion rates, time to target goals, tolerance for over- and undershoot, and safeguards such as sensor failure warnings or redundancy. Long real times can be modeled in fast virtual time.

b Changes in the model can be made quickly. High *N* iterations can be run quickly.

c Our current model responds to fluid dynamics/hydrostatic pressure, similar to a drug with instant on/off features and an exponential dose/response curve. Software may allow modeling of other drug features (e.g., delay the administration of fluid after command given in order to model a delay in drug onset; delay removal of fluid to model delayed offset.).

d Experiments require real time actuation (1:1).

e PID terms can be varied in real time. Different drugs can be tested. Safeguards (e.g., maximum infusion rate) can be tested. Extremes of physiology can be modeled.

f We changed terms during our single experimental session, but sessions may be infrequent and the number of specimens is likely to be low.

g Range of real physiology can be tested, limited by the availability of patient subjects with the condition of interest, clinical and research ethics, and patient safety.

h For reasons of safety, changes in the system require testing before application in humans.

## Methods (general)

All our models used an input value of the current MAP (measured or simulated) and employed a proportional-integral-derivative (PID) control algorithm to modulate an output to control the MAP. PID algorithms are well-tested and have been widely employed for decades in systems such as air conditioning, autopilot and cruise control, and medical automatic insulin pumps (23).

The PID controller is mathematically described by Equation 1:

$$u(t)=K_{\;p}\;e(t)+K_{i}\;\overset{t}{\underset{0}{\int }}\;e(t)dt+K_{d}\;\frac{de(t)}{\;dt}$$ (1)

*e(t)* represents the error, or the difference between the measured MAP and the set point (target value). The PID equation is made up of three components (P, I, and D) that are related to *e(t)* in different ways and are summed together to determine the final output, *u(t)*. In the computer model, *u(t)* was mapped to a simulated drug effect that altered MAP proportionally, with modeled pharmacodynamic properties including onset delay and decay. In the benchtop model, the output was related to the volume of fluid that was infused or withdrawn from the system. In the *in vivo* model, the output corresponded directly to the infusion rate of vasoactive agents.

The proportional (P) term is directly related to the error. The larger the error (larger difference between current MAP and set point), the larger the P term becomes, and the output increases.

The integral (I) term reflects the accumulated error over time. If the MAP remains far from the target, the I term will progressively increase. This allows the controller to increase the output if the system (patient) is less responsive to vasoactive drugs.

The derivative (D) term is directly related to the speed at which the system is approaching or moving away from the target to prevent overshoot or undershoot. For example, if the system is correcting a hypertensive state rapidly, $\frac{de(t)}{\;dt}$ (the rate of change of the error with respect to time) will be a large negative number and therefore will decrease the output to slow the correction and reduce the chance of a significant overshoot.

For each term (P, I, and D), a constant (*K_i_*, *K_p_*, and *K_d_*) can be defined to further tune the algorithm's response to achieve the desired outcome. These constants are weights that determine the magnitude with which each term contributes to the final output. Although initial values can be arbitrarily assigned, the optimal values of each *K* term must be derived through computer models or, preferably, empirically from testing data. The potential range of each constant in a negative feedback system is (0 to ∞). Based on the desired application or response pattern, simpler versions of a PID algorithm can be utilized by setting one or more of the *K* terms to zero, resulting in a P, PI, or PD algorithm.

The PID terms can be optimized for several factors, including speed (time to target), overshoot or undershoot, and stability once the target value is reached. Improving one factor often comes at the expense of another. For example, a large *K_p_* term may allow the system to reach the target more quickly but can also produce significant overshoot. The appropriate balance must be determined for each application.

An optimized PID accurately reaches and maintains the target value at a specified time, along a specified trajectory, with no overshoot and minimal variability. Though not encoded mathematically, in achieving its target, an acceptable PID must also meet other feasibility, reasonableness, and safety constraints (e.g., for a medication infusion, not reaching toxic limits, exceeding tolerable volumes, or inducing unacceptable side effects).

Standard metrics for control system performance include rise time, settling time, and accumulated error (AE) (24). Rise time reflects control system response speed and is defined as the time required to reach a specified percentage of the target value from a specified value (e.g., from 10% to 90%, 5% to 95%, or 0% to 100%). Settling time reflects control system stability and is the time required for the control system to reach and remain within a specified range around the final target value (e.g., ±5%). Accumulated error is the integral of error over time, incorporating both the off-target time and the magnitude of the difference between actual and target values (24, 25). AE is a useful tool for comparing PID parameters when other conditions (initial MAP values, target MAP, and duration of control period) are the same. Other common control system metrics include overshoot, steady-state error (a specific example of accumulated error), and non-conformance or conformance rate. For MAP, on-target percentage (OTP) (conformance rate) is a clinically relevant measure of how reliably a control system maintains the target over time.

The PID controller source code, data, and analysis scripts are available at: https://github.com/ampatel1294/AutomaticControlofArterialPressure.

## Methods (computer model)

We developed a computer simulation of an automatically controlled drug infusion to regulate MAP dynamically and gradually, treating either hypotension or hypertension or maintaining normotension (20, 26). Simulations were implemented in Python 3.7. This approach enables rapid testing and optimization of control strategies and parameters prior to clinical deployment, offering a risk-free, low-cost, and time-efficient framework before physical implementation (Table 2). The simulation modeled hemodynamic responses to a hypothetical drug infusion.

For each simulation, an initial BP was generated and remained stable, in the absence of inputs from the PID controller, with superimposition of Gaussian random noise (mean 0 mmHg, SD 3 mmHg) at each time step. Each simulation lasted 500 cycles [simulated seconds (ss)]; we measured the actual elapsed time as an outcome measure of the efficiency of the research tool, which was influenced by both computer hardware and code efficiency. A control phase was established for the first 100 ss of each case to observe baseline MAP fluctuations. Between 100 and 500 ss, the automatic PID control system was activated and attempted to correct MAP to a target value defined by the operator. As an additional test of control system responsiveness, during some simulations, hypertensive (+20 mmHg) and hypotensive (−20 mmHg) challenges were presented at 200 ss and 300 ss, respectively; each challenge was sustained for 20 simulated seconds, representing sudden substantial changes in a patient's condition, requiring automatic response. We selected PID constants (*K_p_* = 0.3, *K_i_* = 0.014, and *K_d_* = 0.01) based on previously published work, adopting initial published values and iterating through empirical/manual tuning (18). We adjusted the *K_p_* term to provide an appropriate response to the instantaneous MAP error, *K_i_* to respond to the persistent error to target, and then changed *K_d_* based on overcorrection (26). We did not apply systematic methods (e.g., the Ziegler–Nichols method), as our purpose at this stage was not to determine optimal values (27).

In addition to the PID algorithm, we implemented software to limit the rate and to determine the trajectory at which the target BP would be achieved, recognizing that for some clinical conditions (e.g., hypertensive hemorrhagic stroke), overly rapid correction of BP may be deleterious (e.g., inducing secondary end-organ ischemia) (5). For this purpose, we enacted a “moving target” model in which a series of incremental target values were provided to the PID algorithm. We chose an exponential decay curve [*N*(t) = 1e^(−2t)^] to guide the current MAP toward the final target MAP value. We chose this trajectory as a clinically plausible shape that incorporates the biphasic treatment goals recommended for some emergency conditions (Table 1), balancing the need for initial rapid correction of an extreme out-of-physiologic-range BP with more gradual reduction toward a final target. Any trajectory could have been selected, and we did not attempt to determine an optimal trajectory for this simulation. The system was tested in 2,000 simulations with each of five initial MAP values (50, 60, 75, 85, and 95 mmHg) (10,000 simulations total), and again with imposition of hyper- and hypotensive challenges. Time in simulations was represented by ss, where each ss corresponded to one iteration cycle of the PID algorithm. In the computer model, ss are intended to approximate real-time seconds, though the fidelity of this correspondence depends on the accuracy of the underlying pharmacodynamic model.

With the incorporation of the moving target MAP, rise time and settling time converge as similar terms. Consequently, for our primary outcome, we chose OTP versus time from control system initiation to quantify system performance. OTP was calculated as the proportion of simulation runs within the target MAP range at each time point, with 95% Wilson score confidence intervals; rolling-window OTP confidence intervals were calculated using non-parametric bootstrap resampling at the simulation-run level. We also measured the actual time required to complete simulations, an important factor in the utility of a computer model.

## Methods (benchtop model)

A benchtop closed-loop model of fluid resuscitation was built to incorporate and test physical components in preparation for animal testing (Figure 1). This hydrostatic system has limitations (Table 2), including instantaneous onset and offset dynamics that do not represent the delay, half-life, or dose-response saturation of vasoactive drugs; the system was intended to test hardware and software integration and PID response, not to model a specific medication. A 100-mL saline bag represented the patient's circulatory system. The measured hydrostatic pressure of the bag represented the MAP. Two flexible catheters were introduced to the saline bag to (1) measure pressure (representing an arterial pressure monitoring catheter) and (2) add or remove volume [representing an intravenous (IV) infusion catheter], respectively. The arterial catheter and pressure transducer (Edwards LifeSciences Truwave, Irvine, CA, USA) were connected to a medical monitor (GE Dash 4000, GE HealthCare Technologies Inc, Chicago, IL, USA), enabling high-frequency pressure monitoring. The IV line was connected to a custom-built syringe pump that was responsible for fluid volume infusions or withdrawal to adjust the pressure within the vessel. The medical monitor transmitted pressure values at its maximum frequency, once every 5 s, to a microcontroller (Arduino Elegoo UNO R3 ATmega328P microcontroller, Somerville, MA, USA). At each 5 s interval, the syringe pump injected or withdrew fluid based on the output of the PID control algorithm.

**Figure 1:**
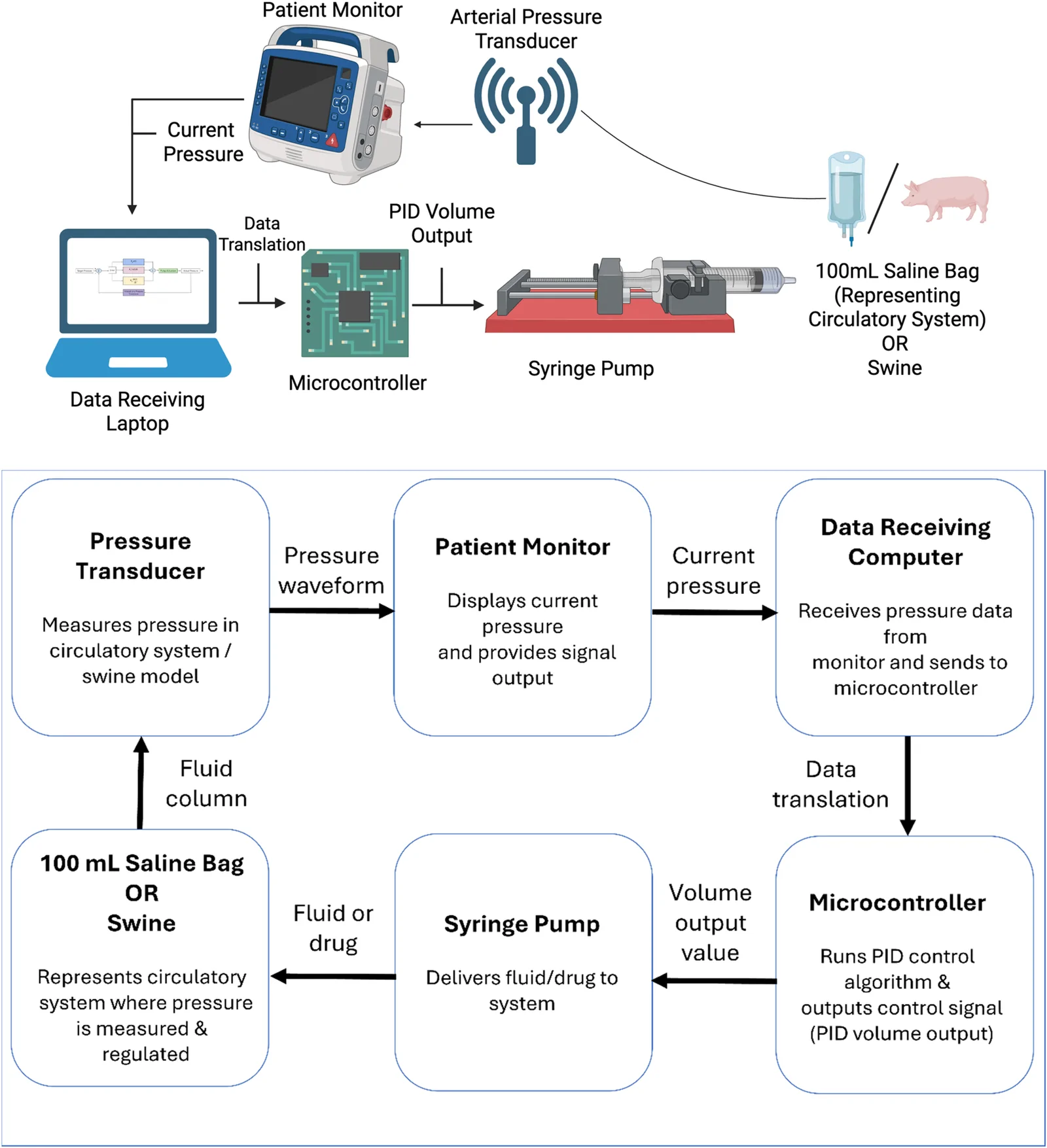
Schematic of the control system used in the benchtop and animal models. *K_p_*, *K_i_*, *K_d_*, and target MAP are set by the operator before each experiment. BP data is transmitted from the transducer to the patient monitor. At 5-s intervals, the MAP is inputted into the PID algorithm along with the set terms. The algorithm output specifies the fluid volume infused or withdrawn from the saline bag (benchtop model) or the vasoactive drug dose administered to a swine (animal model). Created in BioRender. Sriram, R. (2026), https://BioRender.com/4rgt0k9.

Hypotensive and hypertensive rescue and maintenance of an initial on-target pressure were simulated by initializing the saline bag to pressures of 60, 85, 110, 135, and 160 mmHg. For each initial condition, the target pressure was 110 mmHg.

After a 10-s control period (monitoring only), the software was activated to adjust fluid volume within the saline bag until a target MAP equilibrium of 110 ± 5 mmHg was achieved. Three trials were performed at each of the five initial pressures and repeated for P, PI, and PID controllers, resulting in 15 distinct groups and a total of 45 trials.

*K* terms were derived utilizing the Ziegler–Nichols method, which produces distinct optimal constant values for each controller type (P, PI, and PID); consequently, *K_p_* and *K_i_* values differed across the three experiments (27). The trials were performed sequentially for each starting pressure. The benchtop system was completely reset between every individual trial, including recalibrating the pressure transducer. This procedure was used to minimize potential carryover effects from residual fluid volume, catheter position, or system pressure.

We measured rise time (defined as the time required from control system activation until pressure first reached target MAP ± 5 mmHg), settling time (defined as the time from control system activation until the pressure first reached and remained at target MAP ± 5 mmHg until control system deactivation), OTP after rise (defined as the percentage of time from rise until control system deactivation that the MAP was within ± 5 mmHg from the defined target), maximum overshoot (defined as the maximum difference between the measured MAP and target MAP after the target MAP was reached for the first time), and AE after rise [defined as integral of the error (measured from the strict target MAP) over time, from rise time until system deactivation] to quantify the magnitude and duration of excursions from target range and to compare the performance of the system using *K_p_*, *K_i_*, and *K_d_* terms (28). For AE, values were calculated as the integral of error (absolute value of measured MAP minus target MAP) over time, with units of mmHg*s. Two-way ANOVA was used to determine if the performance of the system was affected by altering the constants in the PID model or by altering the starting conditions. *P* < 0.05 was considered significant. Following the ANOVA, Q–Q plots and the Shapiro–Wilk test were performed to evaluate normality of the residuals and the Brown–Forsythe test was performed to assess homogeneity of variance. As the study was not powered to determine which permutation of PID models and starting conditions was optimal, *post hoc* pairwise comparisons were not performed.

## Methods (animal model)

A control system [computer program with a PID control algorithm and physical components (pressure transducer, patient monitor, laptop computer, microcontroller, syringe pump)] (Figure 1) previously tested in a benchtop hydraulic model was applied in the animal model (29). While the automatic controller was active, the algorithm's calculated drug infusion rate was actuated for 4 s, following which 1 s without any infusion was allotted for pressure sampling and subsequent rate adjustment.

The intervention drug was either norepinephrine to raise MAP or clevidipine to lower MAP, administered intravenously. Norepinephrine is a catecholamine vasopressor commonly used for treatment of shock in critically ill patients. It is administered as a continuous intravenous infusion because of its short mean half-life of approximately 2.4 min (30). Clevidipine is an intravenous dihydropyridine calcium channel blocker with rapid onset (2–4 min) and offset (5–15 min) (1, 31). Because of its short half-life (approximately 1 min), clevidipine must be given as a continuous intravenous infusion (28).

### Animals, anesthesia, and standard surgical procedures

A limited animal model (Table 2) was performed as a proof of concept to determine if our system could translate from a benchtop to an *in vivo* model. We used a convenience sample approach, applying our system to automatically control BP in animals already scheduled to undergo non-survival surgical procedures for physician training purposes. All animal procedures were conducted with Institutional Animal Care and Use Committee (IACUC) approval (protocol number: A229-21-11). An amendment was approved for our research to occur concurrently with surgical training (protocol amendment: A229-21-11-24).

Two swine (weight 37.5 kg, 34.5 kg) were placed under general anesthesia for surgical training exercises. The specimens received weight-based ketamine 2.23–3.3 mg/kg intramuscular (IM), acepromazine 1.1 mg/kg IM, buprenorphine 0.01 mg/kg IM, carprofen 4 mg/kg subcutaneous (SQ), bupivacaine 2 mg/kg SQ, and inhaled isoflurane (1%–4%). The animals were monitored throughout the surgical exercises with cardiac rhythm, oxygen saturation, intermittent non-invasive BP, end-tidal exhaled carbon dioxide, and temperature. A left femoral artery catheter was placed under ultrasound guidance in each animal for continuous BP monitoring. A continuous infusion of crystalloid 10 mL/kg/h was administered through a peripheral IV. Ventilator settings were a volume of 7.5 mL/kg, positive end-expiratory pressure (PEEP) of 5 cm H_2_O, rate of 18–22 breaths/min, and end-tidal carbon dioxide (ETCO_2_) of 35–45 mmHg.

Animal #1 was used to test the control system's ability to automatically increase BP to a specified target using norepinephrine (1 µg/mL). The animal was scheduled to undergo a series of surgical interventions for training purposes, which were expected to result in progressively falling BP over time. Surgical procedures were performed over a period of 4 h, including abdominal insufflation with carbon dioxide gas (insufflation pressure of 15 mmHg), laparoscopic gastrocolic anastomosis, open laparotomy, splenectomy, bilateral nephrectomy, and partial hepatectomy. No active or passive warming methods were implemented; the animal was exposed to ambient room air temperatures.

Animal #2 was used to test the control system's ability to automatically decrease MAP to a specified target using clevidipine (0.125 mg/mL). The animal was scheduled to undergo a series of surgical interventions for training purposes, which were not expected to result in significant changes in MAP over time. A right femoral vein catheter was placed under ultrasound guidance for clevidipine infusion. Minimally invasive robotic-assisted laparoscopic surgical procedures (e.g., blunt dissection of the mesentery) were performed over a period of 200 min, including abdominal insufflation with carbon dioxide gas (insufflation pressure of 15 mmHg). The animal was exposed to ambient room air temperatures, with surgical drapes in place. No other warming methods were implemented.

### Euthanasia

Per IACUC-approved protocol, euthanasia was performed at the end of all experimental procedures using 4–10 mmol/kg/IV of KCL under anesthesia as the primary method. Secondary methods to ensure non-recovery included tissue/organ collection and bilateral thoracotomy.

### Interventions (experimental)

For animal # 1 (test of automatic BP augmentation), a series of trials were performed, in each of which a target MAP was set and the PID system was allowed to alter the norepinephrine infusion rate to reach and maintain that target MAP over 10 min, followed by a washout period without norepinephrine for 10 min (four times the norepinephrine mean half-life of approximately 2.4 min) (30). Between trials, refinements to the PID algorithm parameters (*K_p_* and *K_i_*) were made and then tested in the next trial. *K_d_* was 0 throughout. The number of intervention cycles was limited pragmatically by the duration of the surgical training, which ended in euthanasia of the animal as described above. Given the limited number of intervention cycles possible, we chose to explore a range of *K_p_* and *K_i_* values, rather than repeating a single set of values. In addition, replicates with the same *K_p_* and *K_i_* were not performed because the initial conditions of the animal changed between trials as a consequence of ongoing surgical procedures. A maximum infusion rate was applied for safety (to avoid hypertension) and gradually increased with each infusion cycle if the PID algorithm had reached the maximum infusion rate allowed during the prior infusion cycle.

For animal #2 (test of automatic MAP reduction), baseline MAP was measured before infusion of clevidipine. A series of trials were performed, in each of which a target MAP was set and the closed-loop system was allowed to alter the clevidipine infusion rate to reach and maintain that target MAP over 10 min, followed by a washout period of 10 min without clevidipine infusion. Between trials, refinements to the PID algorithm parameters (*K_p_* and *K_i_*) were made and then tested in the subsequent intervention cycle. In addition, for some cycles, a series of incremental target MAPs was implemented to attempt a more gradual reduction to the target MAP.

The results of each trial are shown graphically. For the animal model, statistical inference and comparison between trials could not be made given the sequential, non-independent design; quantities are descriptive only, not inferential. Quantitative assessments of each trial included rise time, settling time, AE after rise time, and OTP after rise time. For MAP augmentation with norepinephrine, rise time was defined as time from system initiation until first MAP value ≥ target MAP −5 mmHg. For MAP reduction with clevidipine, rise time was defined as time from system initiation until first MAP value ≤ target MAP + 5 mmHg. For both medications, settling time was defined as the time at which target MAP ± 5 mmHg was first achieved and then maintained until control system deactivation. If the system did not reach a steady state of target MAP ± 5 mmHg, a settling time was not able to be calculated. AE after rise was defined as integral of the error (measured from the strict target MAP) over time, from rise time until system deactivation. OTP after rise was defined as the percentage of time the MAP was within ± 5 mmHg from the defined target [a variance suggested as acceptable in international sepsis guidelines (8)], from rise time until control system deactivation. Each trial was done sequentially at different stages of a surgical procedure and therefore statistical inference and comparison between trials could not be made.

## Results (computer model)

Figure 2 presents MAP trajectories for two clusters of 10,000 simulations, without and with hyper- and hypotensive challenges. Figure 3 illustrates OTP versus time from system initiation using a rolling 30 ss time window (time t ± 15 ss). Using a tolerance of ± 5 mmHg, OTP reached 90% (Wilson 95% CI 89.4%, 90.6%) at 240 ss and 95% (95% CI 94.6%, 95.4%) at 294 ss and remained at or above these values thereafter until end of experiment. OTP at 100, 300, and 400 ss was 61.0% (Wilson 95% CI 60.0%, 62.0%), 95.5% (95% CI 95.1%, 95.9%), and 99.2% (95% CI 99.0%, 99.4%), respectively. Using a tolerance of ± 2 mmHg, OTP at 100 ss, 300 ss, and 400 ss was 29.0% (Wilson 95% CI 28.1%, 29.9%), 66.7% (95% CI 65.8%, 67.6%), and 80.3% (95% CI 79.5%, 81.1%), respectively. In total, 10,000 regular simulations were completed in 22.57 real minutes (0.135 real s/simulation), while 10,000 simulations with hypertensive and hypotensive events were completed in 25.9 real minutes (0.156 real s/simulation).

**Figure 2:**
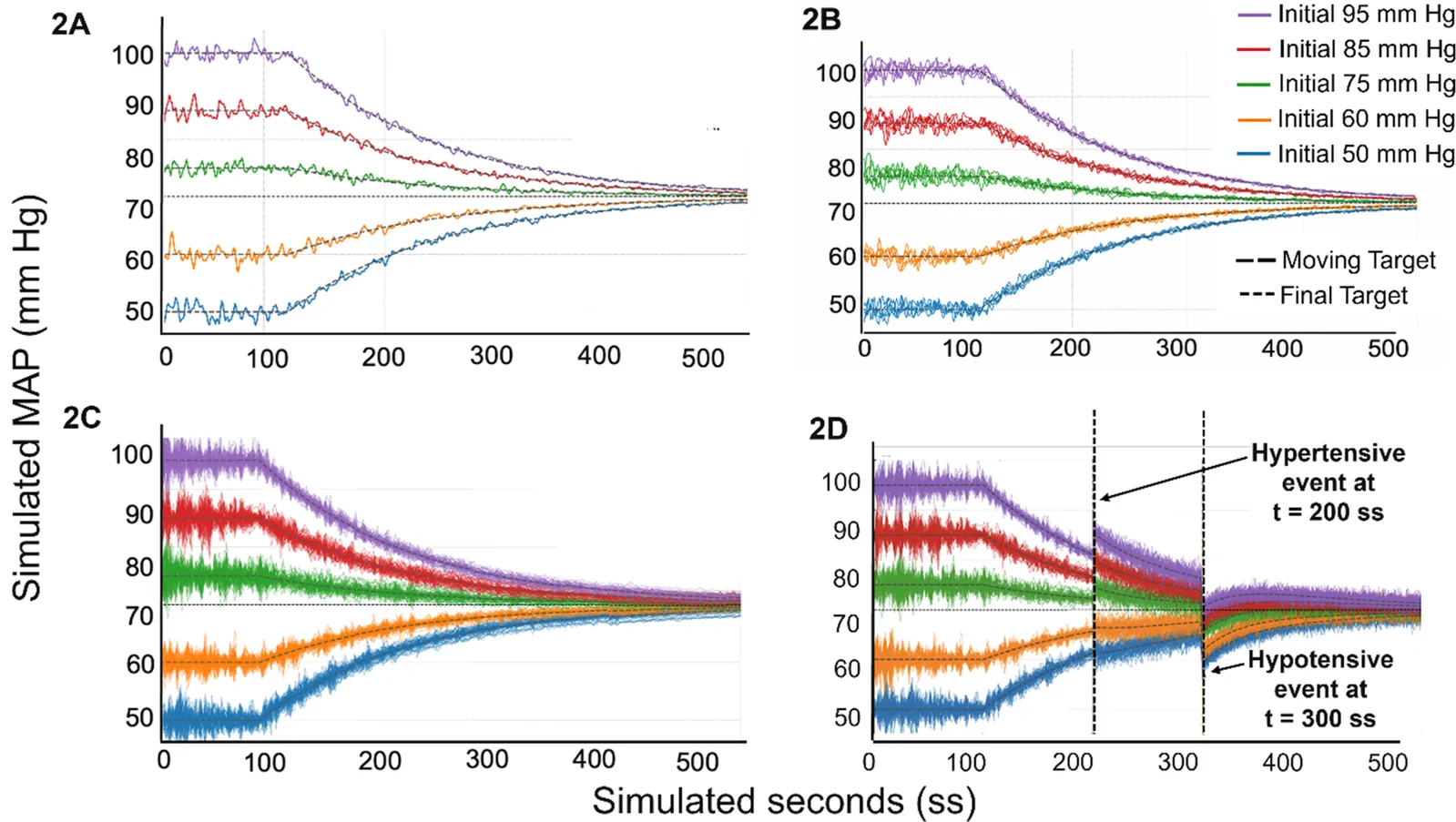
Computer model for automatic blood pressure control. Panel **(A)** shows a single randomly selected sample from 2,000 runs conducted at each of five initial MAPs. The PID algorithm is activated at 100 ss. Each run closely follows the expected exponential decay path calculated for that initial MAP. Panel **(B)** shows five randomly selected samples from each initial MAP. Panel **(C)** shows all 2,000 samples from each initial MAP. Panel **(D)** demonstrates the system's response to hyper- and hypotensive challenges, with the system reasserting control and following a new exponential curve to the target within <30 ss.

**Figure 3:**
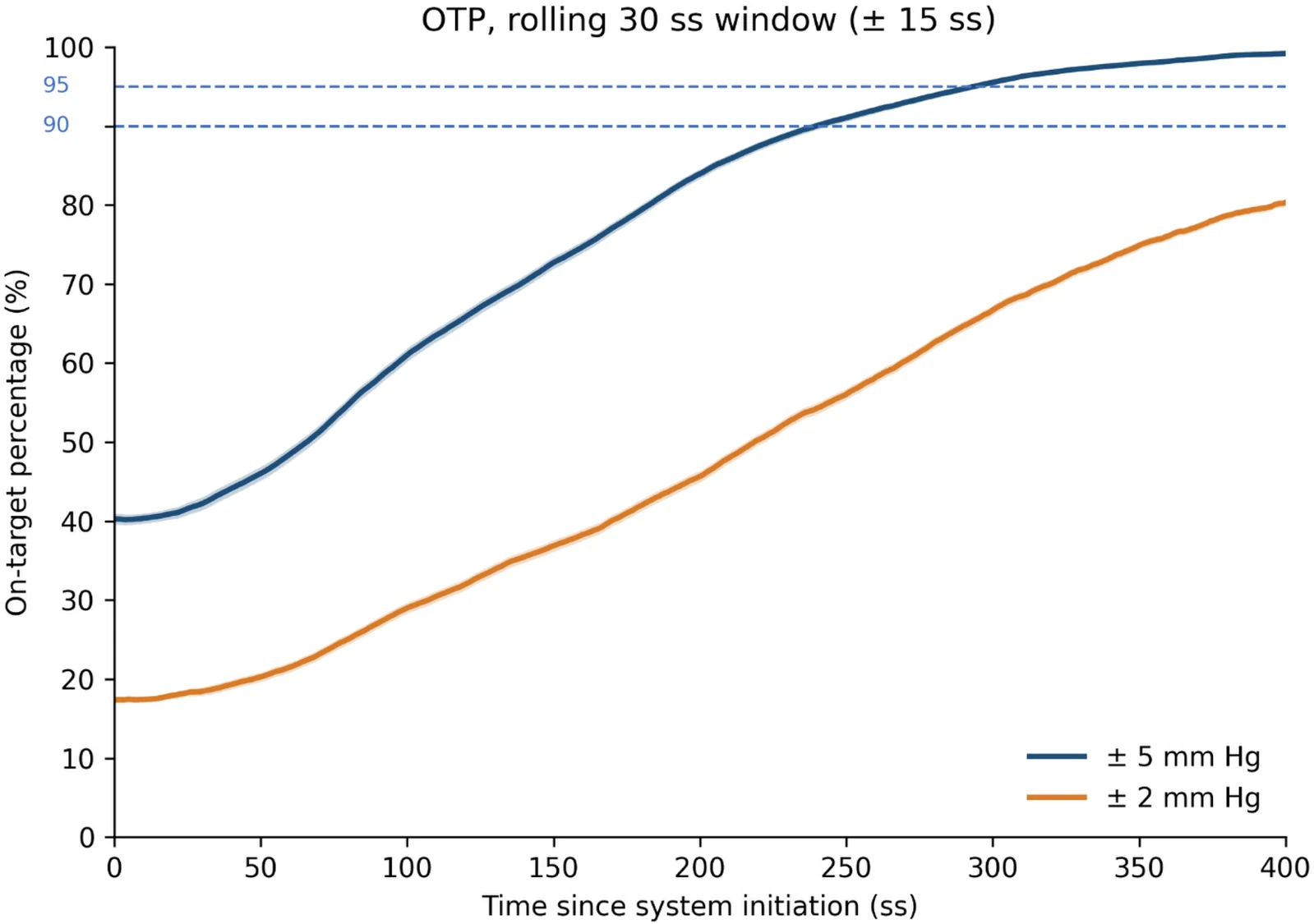
On-target percentage versus time from control system initiation (10,000 samples). OTP values were calculated for a rolling time window of 30 ss (*t* ± 15 ss). OTP was calculated as the proportion of simulation runs within the target MAP range at each time point, with 95% Wilson score confidence intervals; rolling-window OTP confidence intervals were calculated using non-parametric bootstrap resampling at the simulation-run level. Values are shown for two tolerances, target MAP ± 5 mmHg and target MAP ± 2 mmHg, with Wilson 95% CI shaded. For target MAP ± 5 mmHg, OTP climbed steadily, reaching 90% at 240 ss and 95% at 294 ss, and remaining at or above these values thereafter until the end of the experiment. For target MAP ± 2 mmHg, OTP plateaued around 80%. Once the controller locked onto the target, the residual noise floor (introduced intentionally in our model) was approximately 1.6 mmHg, which is close in magnitude to the ± 2 mmHg band itself. As a result, some fraction of runs will always fall just outside that tighter window by chance, regardless of how long the algorithm runs.

## Results (benchtop model)

Figure 4 depicts the change in hydrostatic pressure (a proxy for MAP) with P, PI, or PID controllers, respectively. Figure 5 illustrates rise time, settling time, on-target percentage after rise, and overshoot. Mean rise time with standard deviation was 0.47 ± 0.16 min (P: 0.41 ± 0.05, PI: 0.46 ± 0.10, PID: 0.55 ± 0.24 min; range 0.23–0.96 min) (*p*_controller_ type < 0.001, *p*_initial condition_ < 0.001, *p*_interaction_ < 0.001). All systems reached and maintained equilibrium at the target pressure of 110 ± 5 mmHg. Mean settling time with standard deviation was 0.62 ± 0.18 min (P: 0.59 ± 0.18, PI: 0.56 ± 0.15, PID: 0.70 ± 0.19 min; range 0.32–1.02 min) (*p*_controller type_ = 0.019, *p*_initial condition_ < 0.001, *p*_interaction_ = 0.088). Mean OTP after rise with standard deviation was 91.25 ± 9.68% (P: 89.53 ± 11.92%, PI: 91.44 ± 10.67%, PID: 92.79 ± 6.03%; range 64.22–100.00%) (*p*_controller type_ = 0.71, *p*_initial condition_ = 0.26, *p*_interaction_ = 0.41). Mean AE after rise with standard deviation was 131.42 ± 70.56 mmHg*s (P: 184.95 ± 88.35, PI: 83.98 ± 32.49, PID: 125.33 ± 36.87 mmHg*s; range 28.50–391.11 mmHg*s). Mean overshoot with standard deviation was 3.78 ± 4.84 mmHg (P: 5.67 ± 7.29, PI: 1.17 ± 0.72, PID: 4.50 ± 2.97 mmHg; range 0.00–27.00 mmHg).

**Figure 4:**
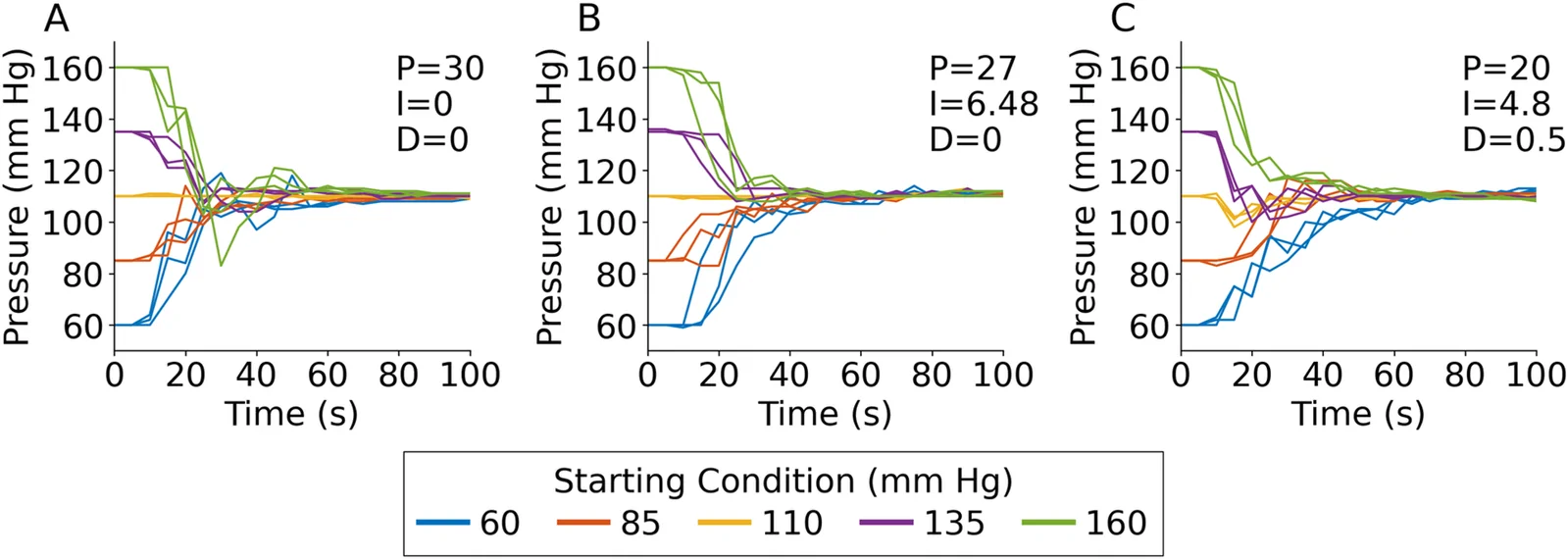
Benchtop model for automatic control of blood pressure. Automatic control of pressure with five unique starting pressures over time. In each experiment, the target pressure was 110 mmHg. Trials were conducted with **(A)** control with P term only (*K_i_* and *K_d_* = 0), **(B)** control with P and I terms (*K_d_* = 0), and **(C)** control with P, I, and D terms. For each initial pressure condition and each control algorithm, three repetitions were performed. In each experiment, the target pressure was rapidly reached and maintained with minimal variability.

**Figure 5:**
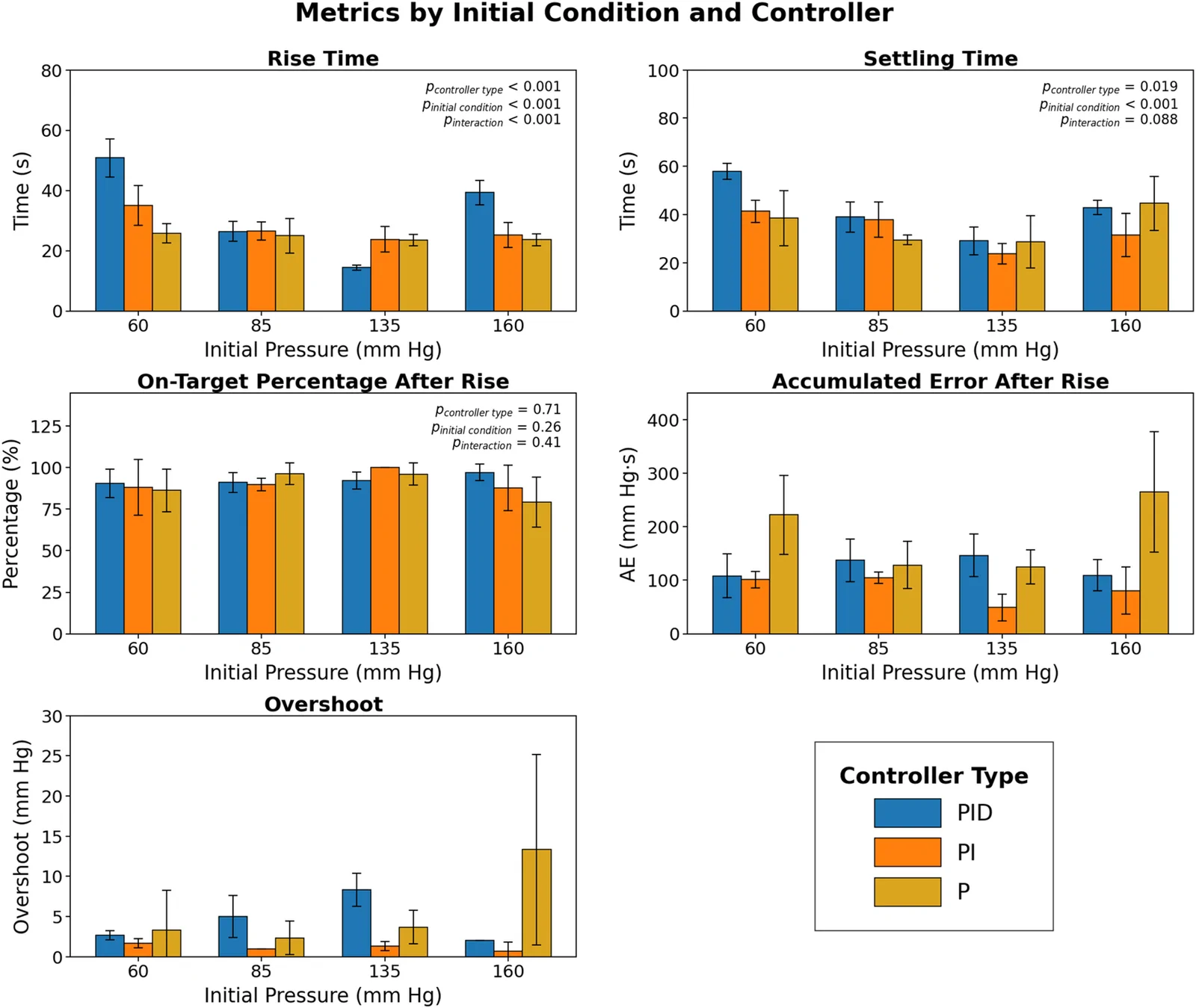
Benchtop model for automatic control of blood pressure. Rise time, settling time, on-target percentage after rise, accumulated error, and average maximum overshoot by initial pressure and controller for the P, PI, and PID controllers. Error bars represent standard deviation. Two-way ANOVA compared whether differences in model type (P, PI, or PID) and initial conditions affect performance metrics. For AE and overshoot, *p*-values are not reported as data were not normally distributed.

We evaluated the assumptions underlying the two-way ANOVA model. Residual Q–Q plots and Shapiro–Wilk tests demonstrated approximate normality for rise time, settling time, and OTP after rise. AE after rise (*p* = 0.010) and overshoot (*p* < 0.001) were not normally distributed; consequently, *p*-values for AE and overshoot were omitted and the analysis should be considered only exploratory. Brown–Forsythe tests demonstrated homogeneity of variance in all the groups (all *p* ≥ 0.589).

## Results (animal model)

### Automatic blood pressure augmentation with norepinephrine

Figure 6 depicts MAP in swine specimen #1 during automatic PID algorithm BP augmentation with norepinephrine infusion, each cycle preceded and followed by periods without norepinephrine. Rise time, settling time, on-target percentage after rise, and accumulated error after rise are also reported for each trial in Figure 6.

**Figure 6:**
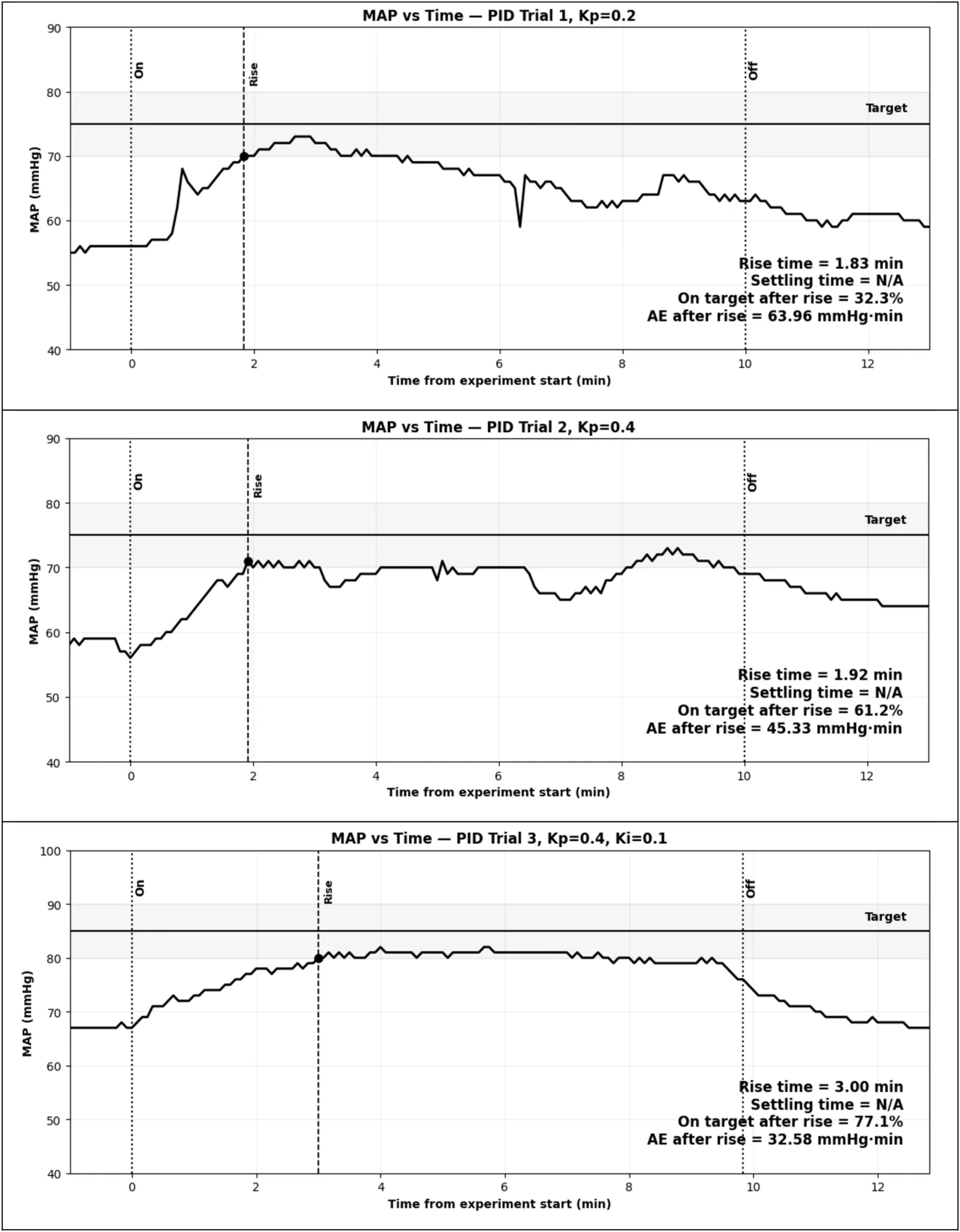
Automatic blood pressure control with norepinephrine over a 2.5-h period in a swine undergoing surgical interventions. PI terms (*K_p_* and *K_i_*) are indicated; if not listed, term = 0. Rise time, settling time, on-target percentage after rise time, and accumulated error after rise time are shown for each trial. On (dotted line) indicates system activation. Off (dotted line) indicates control system deactivation. Rise (dashed line) indicates rise time. Target (solid line) indicates the setpoint MAP value. Gray shaded regions indicate target MAP ± 5 mmHg.

Mean rise time was 2.25 min (range 1.83–3.00 min, SD 0.65 min). No trial maintained an equilibrium value of target MAP ± 5 mmHg; therefore, no settling time could be calculated. Mean OTP after rise was 56.89% (range 32.32–77.11%, SD 22.71%). Mean AE after rise was 47.29 mmHg·min (range 32.58–63.96 mmHg·min, SD 15.78 mmHg·min).

### Automatic blood pressure reduction with clevidipine

Figure 7 depicts MAP in swine specimen #2 during automatic PID algorithm BP reduction with clevidipine infusion, each cycle preceded and followed by periods without clevidipine. A single target MAP was applied for each cycle. Mean rise time was 1.00 min (range 0.50–1.44 min, SD 0.40 min). Mean settling time was 2.19 min (range 1.17–4.33, SD 1.45). Mean OTP after rise was 93.8% (range 87%–100%, SD 7.18%). Mean AE after rise was 12.64 mmHg·min (range 10.54–15.57, SD 2.30).

**Figure 7:**
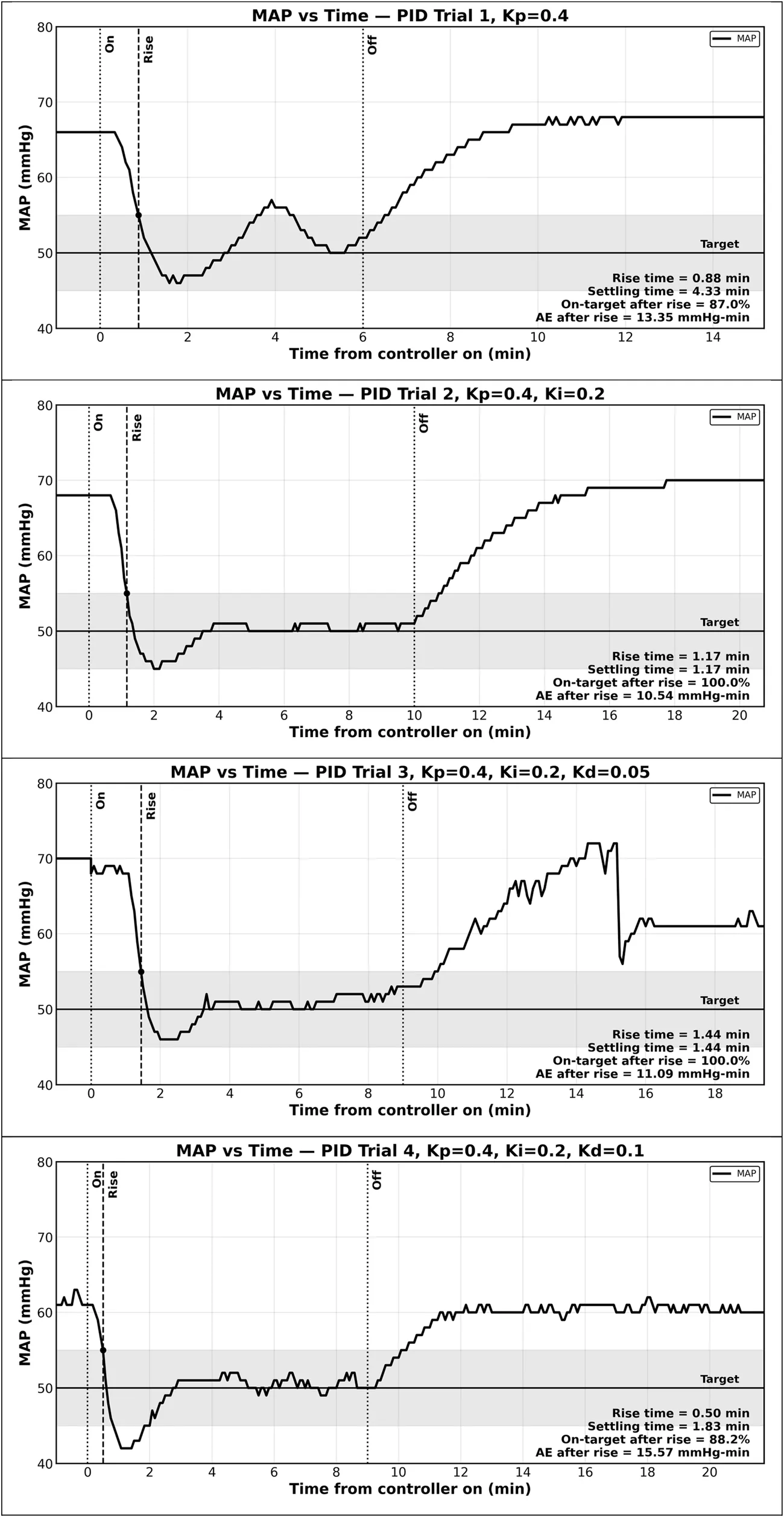
Automatic control of blood pressure using clevidipine in a swine, using a single final BP target. PI terms (*K_p_* and *K_i_*) are indicated; if not listed, term = 0. Rise time, settling time, OTP after rise, and AE after rise are shown for each trial. On (dotted line) indicates system activation. Off (dotted line) indicates control system deactivation. Rise (dashed line) indicates rise time. Target (solid line) indicates the setpoint MAP value. Gray shaded regions indicate target MAP ± 5 mmHg.

Figure 8 depicts automatic BP augmentation using clevidipine in swine #2, with stepped incremental BP targets to slow the rate of change and time to final target MAP. Trial 5 used 2 mmHg target decrements from 60 to 50 mmHg, Trial 6 used 2 mmHg target decrements from 62 to 50 mmHg, Trial 7 used 4 mmHg target decrements from 62 to 50 mmHg, and Trial 8 used 4 mmHg target decrements from 60 to 48 mmHg. Because the initial interval target was the same as current MAP, rise time was (by definition) 0.00 min for all stepped target clevidipine trials. Mean settling time was 2.79 min (range 0–4.67 min, SD 2.12 min). Mean OTP after rise was 85.85% (range 77.9%–100%, SD 9.94%). Mean AE after rise was 30.39 mmHg·min (range 20.71–37.92 mmHg·min, SD 8.15 mmHg·min).

**Figure 8:**
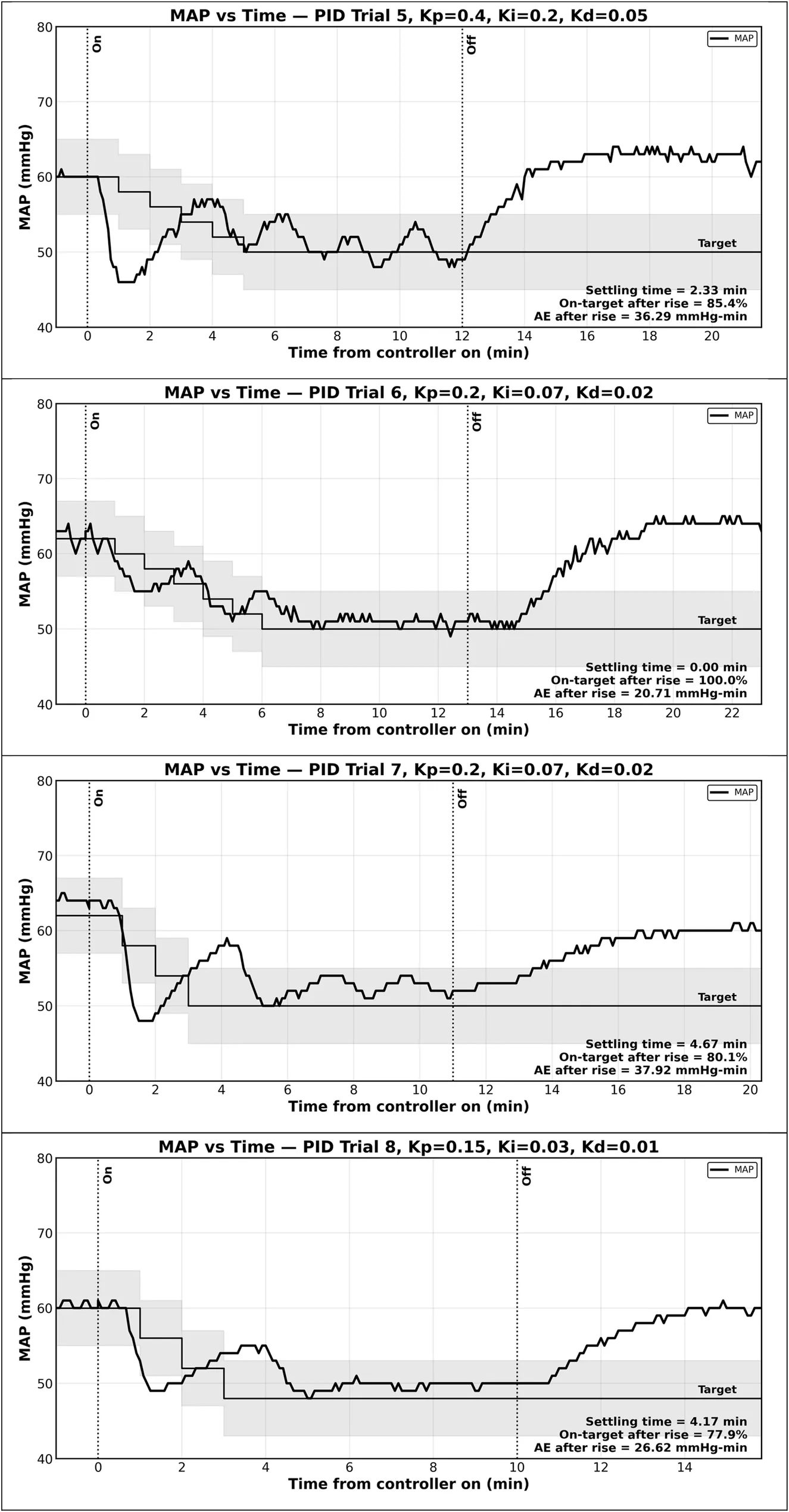
Automatic control of blood pressure using clevidipine in a swine, using stepped/interval blood pressure targets (programmed target MAP decrements of 2 or 4 mmHg every minute) toward a final target MAP. PI terms (*K_p_* and *K_i_*) are indicated; if not listed, term = 0. Rise time, settling time, OTP after rise, and AE after rise are shown for each trial. On (dotted line) indicates system activation. Off (dotted line) indicates control system deactivation. Target (solid line) indicates the setpoint MAP value. Gray shaded regions indicate target MAP ± 5 mmHg.

## Discussion

In this proof-of-concept study, we implemented an automatic PID algorithm for BP control across computer, benchtop, and swine models. Each model provides unique limitations and benefits for further refinement of the system (Table 2).

Computer models allow rapid and inexpensive iteration of large numbers of cases to explore a range of values for PID parameters and trajectories, at the expense of realism. Computer models can be further refined to represent different medications (e.g., onset, half-life/duration, and amplitude of effect), patient phenotypes (e.g., non-responders, average responders, and super-responders), disease processes (e.g., hypertensive hemorrhagic stroke and septic shock), target values (e.g., MAP, systolic BP, and diastolic BP), or dynamic physiology [e.g., a patient who becomes progressively sicker (e.g., hypothermic and acidotic) and therefore becomes progressively less responsive to medications, requiring higher doses to achieve the desired effect]. Benchtop modeling allows testing of computer models with physical equipment *en route* to *in vivo* testing, with intermediate time costs and economic expenses. *In vivo* testing in animals is the most realistic platform prior to human testing, allowing confirmation of PID algorithm with real pharmaceuticals and physiology, but it is expensive, time consuming, and logistically complex, with less opportunity for large sample sizes. Together, the platforms offer a synergistic set of tools.

The performance of the algorithm in three different settings with minimal tuning supports the robustness of this approach. In benchtop testing, we used fluid boluses, which have immediate onset and offset of pressure effects (resembling a medication with instantaneous onset, sustained effect, and immediate offset/reversal ability). These effects are not true of most pharmaceutical agents, for which delay in onset, variable persistence of effect due to biological half-life, and reduced magnitude of effect at high doses may be encountered. Despite these differences, we were able to empirically adapt PID terms to allow the algorithm to approach target MAP in the swine model using norepinephrine and clevidipine. These agents share rapid onset and offset, characteristics that explain their preferred use in critically ill patients with rapidly changing/volatile BP and make them ideal for an automatic control system. Agents with slower onset and offset would be more challenging, though not impossible, to adopt in a control system; for example, heating and cooling systems for homes have relatively slow response times yet are widely used.

The proof-of-concept animal data demonstrate that an automatic control system has the potential to regulate arterial BP *in vivo* under dynamic, clinically realistic conditions. In the first animal model, the system was able to infuse norepinephrine to augment the MAP across several combinations of *K_p_* and *K_i_* terms. The control system did not reach the target MAP nor settle; this may have been the result of the extreme physiological conditions, which included hypothermia, an open abdomen with evisceration of organs, active surgical manipulation, and hemorrhagic shock (best treated with blood transfusion, not vasopressor support). Trial 3 showed close adherence to the goal MAP range, with only transient and low-amplitude deviations after rise (Figure 6). Future trials can employ a more accurate animal model of vasodilatory shock (amenable to vasopressor therapy). While improvements in the algorithm are also likely possible, even an imperfect automatic control system could have clinical value by rapidly responding to changes in patient condition, blunting deterioration, and alerting human providers to act.

Overall, automatic clevidipine infusion rapidly reduced MAP to the target range with minimal accumulated post-rise error across the tested PID configurations. In the second animal model, the system reduced MAP with clevidipine in all trials, rapidly reaching the target MAP and maintaining the target range. The addition of stepwise interval targets allowed the rate of pressure change to be controlled. The studies were not powered or designed to determine the optimal terms for the model. However, the two experiments showed that it is technically feasible for the automatic control to alter the infusion of vasoactive drugs in a clinically relevant way. Future studies are needed to systemically assess a range of PID terms under controlled and repeated initial conditions to optimize the PID terms. Our study also demonstrated how metrics such as rise time, settling time, OTP, and AE can be calculated and can be used in future studies to compare different iterations of the PID model.

In none of our models did we enter drug-specific information, again suggesting that the system may be robust enough to appropriately modulate BP across a wide range of initial conditions and clinical scenarios. Varying the *K_p_*, *K_i_*, and *K_d_* terms could allow even more accurate control of BP for specific medical conditions, medications (and concentrations), and patient factors. For example, the optimal values of each term may be very different for medications with differing onset, half-life, and peak magnitude of effects. The desired time to target BP (rise time and settling time) may be different in a hypertensive emergency [where prior studies show an association between rapid correction and adverse outcomes (4, 5)], compared with hypotensive emergencies where an immediate correction to a minimum perfusing BP is likely essential while avoiding overshoot (8, 9, 14). In addition to the PID algorithm, other software controls could be implemented to allow users to determine the rate at which the target BP is achieved, the relative “memory” of the adaptive response (modeled in the I term, which reflects the accumulated error over time), and even the “shape” of the trajectory from initial BP to target (versions of which we implemented in our computer and animal models, e.g., exponential decay or stepped) (32, 33).

In addition to potential clinical uses, a PID controller that allows accurate, precise, and stable BP regulation with customizable time-to-target would be an invaluable research tool to test therapeutic interventions and establish best therapeutic targets. Past research has been limited by the inability to reach and maintain BP targets reliably to distinguish between treatment groups (3). Our system measured and controlled MAP, while some guidelines reference systolic and diastolic BP. It would be simple to modify the system to measure and control these parameters. In addition to controlling BP, PID systems could target other individual physiological values or combinations of parameters, such as cerebral oxygen delivery or cerebral perfusion pressure.

### Limitations

We did not yet systematically investigate the optimal P (*K_p_*), I (*K_i_*), and D (*K_d_*) terms for any of our models through theoretical or empirical means. Systematic tuning may improve performance, although it is unlikely that a single set of terms is “optimal” for all clinical scenarios. The objective of this study was to demonstrate that an automated approach to titrating vasoactive infusion was technically possible and to determine viability for clinical translation. Further work is required to tune the PID system for specific clinical applications. In addition, it may be possible to create a self-tuning system that optimizes each term based on real-time performance. We have not yet compared the performance of automatic PID control to the performance of human operators, an important topic for future research. Before this can be translated to the clinical setting, future work is needed to compare the efficacy and safety of the system compared to manual titration, establish safeguards in the algorithm, and determine the appropriate clinical indications.

In our benchtop model, AE and overshoot showed significant departures from normality, limiting the suitability of two-way ANOVA inference, which assumes normality of data. Consequently, the *p*-values for AE and overshoot were omitted and the analysis should be considered only exploratory.

Our animal study utilized only two swine specimens, and we were unable to repeat experimental cycles under identical conditions, given that we were pragmatically testing concurrently with surgical training exercises. Our outcomes may have been different with a greater number of specimens (perhaps with different genotypes and phenotypes with variable responses to norepinephrine and clevidipine, or greater propensity to hypo- or hypertension). This approach was sufficient for a proof-of-concept, but future studies will need to be powered adequately to determine the optimal parameters of the system.

The specimen used to test BP augmentation with norepinephrine likely had hemorrhagic shock, which would be best managed with blood transfusion, not vasopressor support. Our clevidipine trials lowered BP from an already normotensive value. Future studies should include additional hypotensive and hypertensive scenarios to test the system robustness under various clinically relevant physiological conditions.

#### Future directions: regulatory and engineering safety considerations

While our data suggest that this automated approach to controlling BP is technically feasible, additional experimental validation, regulatory steps, and engineering measures are required before such a system could be safely used in clinical practice. Engineering considerations include hardware, software, and human factors. We consider some of these issues in the context of vulnerabilities of current human-directed clinical practices and devices, as well as existing high-risk control devices.

The need for highly reliable safety measures is not unique to our proposed system; closed-loop controllers have been implemented in existing high-risk medical devices such as insulin pumps (34) and automatic implantable cardioverter-defibrillators and pacemakers (35), where device failure (including over- and undertreatment) could be fatal. Moreover, while end-users of electronic medical devices may perceive these as primarily hardware, all such devices rely on onboard software [Software in a Medical Device (SiMD)], subject to the same types of risks as our system. For example, pulse oximeters are subject to errors in patients with darker skin pigmentation (calibration errors), leading to under-recognition and undertreatment of hypoxia (32). Automatic non-invasive BP cuffs can misread BP due to software or hardware errors; a commonly recognized error is under- or over-cuffing, in which an inappropriately small cuff results in erroneously high BP readings, or an inappropriately large cuff results in erroneously low pressure readings, either of which could result in mistreatment (33). Outside of medicine, self-driving/autonomous vehicles are yet another immediate example of high-risk implementation of automated control systems. The risk of a device-related adverse outcome must be weighed against the risk of adverse outcomes in its absence.

##### Foreseeable software and hardware failures

All modern electronic medical devices are subject to hardware and software failures, ranging from frank non-functionality (e.g., electrical short and power failure) to malfunctioning (e.g., continued but aberrant function, such as a programmable pump delivering the wrong quantity of medication). In a closed-loop BP control system, errors in any of the system components (Figure 1) could result in dangerous failure of the system. Notably, these same risks already apply to existing human-guided BP control (which shares many of the same components), such as the BP pressure transducer, patient monitor, and programmable infusion pump.

##### BP transducer/sensor errors

Sensor errors resulting in erroneously high or low BP readings could pose significant patient risk, as these in turn would lead to algorithmic treatment errors by an automatic control system. Invasive arterial catheters and transducers must be properly placed and calibrated. A catheter that is positioned against a blood vessel wall may give a dampened signal, often recognized only by astute clinicians who perceive the arterial waveform to be unexpected in its shape or the numerical values it provides. A miscalibrated transducer can provide wrongly high or low BP readings; this scenario can result from initial calibration errors or a change in a patient's position after an initial correct calibration step. Again, human operators are often the only current safeguard against this form of error. Clinicians may recognize a disconnect between the patient's condition and the measured BP value (e.g., a patient whose BP appears critically abnormal while the patient appears normal, or vice versa). Redundancy offers a potential remedy and is commonly used for critical systems throughout engineering. Notably, the current clinical standard does not include robust redundancy; a non-invasive (and potentially inaccurate) BP cuff reading is commonly used as an intermittent comparator for the invasive BP reading. It is not currently common clinical practice to place a second invasive BP catheter and transducer; even in the rare instances in which this is done (e.g., dissection of the aortic arch), BP readings from the two devices may be different because of actual BP differences between the vascular beds into which they are inserted. Future solutions for closed systems may include redesigned invasive catheters that allow redundant BP readings from multiple ports on the same intravascular device or redundant calibration steps.

Real-world data addresses the frequency of these hypothetical concerns. Faulty BP data from invasive arterial monitors can result from multiple causes, including damping (loss of energy as the BP pulse wave is conducted through a fluid medium to the electronic transducer); transducer leveling and calibration (zeroing) errors; movement artifacts; calibration drift; and mechanical factors such as catheter occlusion, kinking, and dislodgment. Underdamping has been reported in 30.7% of cardiovascular surgical patients, with systolic BP values overestimated by an average of 28.5 mmHg (36). Overdamping results in erroneously low systolic BP values (37). Failures attributed directly to faulty transducers, monitors, cables, and related components appear rare. A study of transducers in an intensive care setting found transducer readings to accurately match reference standards (38). In a voluntary anesthesia adverse event reporting database spanning 10,518 events from 2009 to 2023, only 27 events (0.26%) were attributed to invasive arterial pressure equipment failure (37). Of these, six (22%) were attributed to inappropriate transducer height (not malfunctioning equipment) causing erroneous BP values, including one case with unrecognized hypotension and multiorgan failure (37).

##### Pump malfunction

Pump malfunction is an important potential risk with a closed-loop control system, as well as a critical risk in existing human-operated systems. Pump malfunction spans hardware, software, and human factor vulnerabilities. Existing programmable (smart) pumps can lose power, have failure of mechanical or electronic components, experience failure from occlusion of attached tubing (e.g., commonly from kinking of tubing from patient movements, especially in upper extremity peripheral IVs), or simply run out of the infusing medication. Current systems can alarm for some of these states (e.g., emitting an audible tone, sometimes accompanied by a text message locally on the device, such as a power or occlusion warning). These alarms require a clinical provider to be locally present and recognize and address the error; systems do not routinely contact providers remotely through means such as automated text or audio messaging, although such solutions could be implemented. Alarm management for an automatic system could also include automatic reversion to a “safe mode,” such as a fixed infusion rate within designated limits, accompanied by strident alarms to patient care providers.

Current clinically deployed programmable pumps routinely include software limits on maximum dose and infusion rates, sometimes called “guardrails.” In the past, these were manually programmed by users; today, many systems have preprogrammed limits specific to the medication being infused, with the user simply selecting the appropriate drug. Even such systems are subject to errors, such as user error in selecting the wrong drug (therefore resulting in incorrect infusion rates and limits), user override of limits, programming errors (e.g., wrong units used for a drug, leading to dosing errors), or the drug loaded into the pump being incorrect or of an unexpected concentration (39). The same types of solutions required to protect against these error conditions with human operators and programmable pumps would be required for a closed-loop system.

Of these error sources, human factors appear most common. In one multicenter study, errors occurred in 60% of smart pump infusions, including operators bypassing or overriding pump guardrails (preprogrammed libraries of infusion rate/dose limits) or selecting incorrect concentration, dose, or rate of drug administration. While the majority of errors did not lead to patient harm, such errors could be fatal in the setting of vasoactive agents in an already unstable patient (40). A systematic review of smart pumps found that although reduction in errors occurred with their use, a variety of errors were not prevented, such as selection of the wrong drug, non-compliance with (overriding of) drug libraries/guardrails, errors in the drug library (e.g., programmed doses are incorrect), and lack of programming for titratable drugs (41). A systematic review of the precision and reliability of infusion pumps found gaps in assessment of both (42). Pump hardware failures appear uncommon; in one study, such failures occurred at a rate of 70/15,052 (0.465%), including overflow (0.001%), underflow (0.0015%), display errors (0.0018%), and cracked infusion system (0.0003%) (43).

##### Regulatory considerations

Our proposed system necessarily acts in concert with other hardware and software components (pressure transducers, patient monitors, infusion pumps, and their associated computer code) and therefore would be defined as software that drives or controls a medical device, i.e., SiMD, not Software as a Medical Device (SaMD), under current United States Food and Drug Administration (US FDA) regulations (44). Manufacturers could choose to integrate closed-loop automation features fully or to allow third-party add-on integration. The European Union Medical Device Regulation (EU MDR) categorizes both SaMD and SiMD as Medical Device Software (MDSW). Other regulations may be in place around the world.

##### Target population

As we have previously outlined (Table 1), many diverse medical conditions may benefit from automatic BP control. Target populations could include patients with cardiovascular and neurological emergencies and shock states, as well as patients undergoing elective procedures in which scripted control of BP could be essential (e.g., elective cardiovascular surgical or endovascular procedures). While careful validation of the performance of an automatic system in multiple clinical conditions is warranted, predicates exist for general-use critical care tools, such as a ventilator with automated settings for management of a wide range of critical conditions requiring respiratory support [e.g., FDA 510(k) document states: “[the device] is intended to provide continuous ventilation for patients requiring respiratory support. This product is intended for a wide range of patients from infant to adult and for a wide variety of clinical conditions.”] (45).

##### Clinical setting

The most obvious initial high-demand clinical settings in which automatic BP control systems may be deployed are emergency departments, intensive care units, and operating/surgical theaters, substituting for existing human-dependent BP control. Once properly validated, the range of possible use settings could be expansive, including prehospital/interhospital critical care transport (where critical patients may require tight BP control despite minimal available staff, who may also be less-trained caregivers); military installations and long-range military transport; other austere locations on Earth (e.g., scientific outposts in Antarctica); and in space, where future missions beyond low Earth orbit may require on-board care with scant personnel. From a regulatory perspective, clearances for more than one clinical setting can occur (45).

##### Intended operator

The intended operators of automatic BP control systems would initially include the same critical care personnel currently managing BP in acute care patients, such as physicians, nurses, and pharmacists. As systems are validated, the user base may further expand to include physician assistants, paramedics, and others. While such a wide range of end-users demands careful design of human interfaces and automated safeguards, these demands have been fulfilled for other high-risk clinical systems. Paramedics deploy automatic chest compression devices and defibrillators; pharmacists and nurses operate programmable infusion pumps (with the caveat that errors are known to occur despite safeguards); respiratory therapists, physicians, physician assistants, and critical care nurses operate ventilators; and patients operate insulin infusion pumps, responding to alerts and servicing the systems by replacing medications and other infusion components. Closed-loop insulin delivery systems are associated with higher OTP than open-loop systems, as well as lower rates of hypoglycemic coma (overtreatment). Hyperglycemia risk/diabetic ketoacidosis (undertreatment) rates are higher (though rare) with closed-loop systems, attributed to infusion set malfunction rather than PID algorithm errors (34).

##### Degree of human oversight and conditions under which human intervention would override the automated controller

Automatic BP control is likely most necessary and beneficial in patients with critical physiology (whether from an emergency or in the setting of a planned but sensitive invasive medical procedure). Consequently, human providers are likely to be immediately available to provide oversight. Systems should be designed both for high automaticity and with the ability for human supervision and override. The purpose of an automatic system is not necessarily to entirely obviate human oversight but to allow human attention and expertise to be turned to other cognitive and physical tasks, with immediate or rapid ability to take control when needed. Aids to this deployment include means of informing operators of the system state with indicators of normality/nominality (as in astronautics, meaning all systems performing as planned) and clear and specific alerts for non-nominal or unexpected conditions (e.g., unexpected BP values or drug doses) (46). The conditions under which human operators would override the automated controller would include frank failures of the system (such as loss of power or system warnings of component failures) and more subjective circumstances, such as the system failing to achieve programmed BP goals (which may be the result of patient pathophysiology or of a technical failure of the control system). Human critical care providers are already accustomed to intervening in such circumstances, such as disconnecting patients from automatic ventilators and providing manual ventilation to eliminate a complex “black box” system and reduce the risk of an unrecognized technological failure.

The degree of human oversight required will likely change over time with improvements in technology. Autonomous driving systems provide an example: early systems required an onboard human driver equipped with a steering wheel, ready to take control at any time. Later iterations allowed remote human operators to intervene. Current generations include fully autonomous systems. Outcomes with autonomous and human driving systems also provide a comparator for BP control systems; while autonomous driving systems have a non-zero rate of accidents, including those with injury and fatality, these must be considered in comparison to rates with human drivers, with some studies showing substantially better outcomes (55% reduction in crash rate, 80% reduction in injury) with automation (47).

##### Cybersecurity

Cybersecurity is essential in all modern electronic medical (and non-medical) products deployed in high-risk scenarios; this is not unique to a BP control system and has been recognized for more than a decade (48). A programmable medication infusion pump could be hacked remotely or locally, resulting in potentially fatal over- or undertreatment of a patient. The famed Stuxnet hacking of Iranian centrifuges, causing physical damage, could potentially be replicated with an infusion pump (49). Innumerable other risks can be imagined: an electronic monitor could be hacked to provide erroneous physiologic data, leading to patient mismanagement. An AICD/implanted cardiac pacemaker could be maliciously programmed to shock a patient incessantly, to fail to pace, or to overheat or deplete the battery prematurely (48). Thus, validated cybersecurity measures should be mandatory for both existing devices and any clinical implementation of a closed-loop BP control system.

Given these considerations, validation requirements for a clinical system must be numerous and robust, but the successful deployment of other similarly sensitive products provides hope and a roadmap. The U.S. FDA publishes detailed guidance documents (50, 51).

## Conclusion

These proof-of-concept studies utilizing computer, benchtop, and animal models provide initial evidence that an automatic PID algorithm has potential to control BP across a range of starting BP. Future studies can optimize the system and compare its performance to human operators in advance of potential human clinical trials.

## Data Availability

The raw data supporting the conclusions of this article will be made available by the authors, without undue reservation.

## References

[1] Peixoto AJ. Acute severe hypertension. N Engl J Med. (2019) 381(19):1843–52. 10.1056/NEJMcp1901117 31693807

[2] Whelton PK Carey RM Aronow WS Casey DE Collins KJ Dennison Himmelfarb C. 2017 ACC/AHA/AAPA/ABC/ACPM/AGS/APhA/ASH/ASPC/NMA/PCNA guideline for the prevention, detection, evaluation, and management of high blood pressure in adults: executive summary: a report of the American College of Cardiology/American Heart Association task force on clinical practice guidelines. Hypertension. (2018) 71(6):1269–324. 10.1161/HYP.0000000000000066 29133354

[3] Anderson CS Heeley E Huang Y Wang J Stapf C Delcourt C. Rapid blood-pressure lowering in patients with acute intracerebral hemorrhage. N Engl J Med. (2013) 368(25):2355–65. 10.1056/NEJMoa1214609 23713578

[4] Peacock F Amin A Granger CB Pollack CV Levy P Nowak R. Hypertensive heart failure: patient characteristics, treatment, and outcomes. Am J Emerg Med. (2011) 29(8):855–62. 10.1016/j.ajem.2010.03.022 20825913

[5] Mayer SA Kurtz P Wyman A Sung GY Multz AS Varon J. Clinical practices, complications, and mortality in neurological patients with acute severe hypertension: the Studying the Treatment of Acute hyperTension registry. Crit Care Med. (2011) 39(10):2330–6. 10.1097/CCM.0b013e3182227238 21666448

[6] Katz JN Gore JM Amin A Anderson FA Dasta JF Ferguson JJ. Practice patterns, outcomes, and end-organ dysfunction for patients with acute severe hypertension: the Studying the Treatment of Acute hyperTension (STAT) registry. Am Heart J. (2009) 158(4):599–606 e1. 10.1016/j.ahj.2009.07.020 19781420

[7] Vuylsteke A Vincent J-L Payen de La Garanderie D Anderson FA Emery L Wyman A. Characteristics, practice patterns, and outcomes in patients with acute hypertension: European registry for Studying the Treatment of Acute hyperTension (Euro-STAT). Crit Care. (2011) 15(6):R271. 10.1186/cc10551 22087790 PMC3388647

[8] Prescott HC Antonelli M Alhazzani W Møller MH Alshamsi F Azevedo LCP. Surviving sepsis campaign: international guidelines for management of sepsis and septic shock 2026. Crit Care Med. (2026) 54(4):725–812. 10.1097/CCM.0000000000007075 41869847

[9] Hirsch KG Amorim E Coppler PJ Drennan IR Elliott A Gordon AJ. Part 11: post-cardiac arrest care: 2025 American Heart Association guidelines for cardiopulmonary resuscitation and emergency cardiovascular care. Circulation. (2025) 152(16_suppl_2):S673–718. 10.1161/CIR.0000000000001375 41122894

[10] Sperry JL Martin MJ Moore EE Sava JA Ciesla D Rizzo AG. Prehospital resuscitation in adult patients following injury: a Western Trauma Association critical decisions algorithm. J Trauma Acute Care Surg. (2019) 87(5):1228–31. 10.1097/TA.0000000000002488 31464868

[11] Jentzer JC Burstein B Van Diepen S Murphy J Holmes DR Bell MR. Defining shock and preshock for mortality risk stratification in cardiac intensive care unit patients. Circ Heart Fail. (2021) 14(1):e007678. 10.1161/CIRCHEARTFAILURE.120.007678 33464952

[12] Chen JT Roberts RJ Sevransky JE Gong MN. Resuscitation in the first 3 hours of sepsis-induced hypotension varies by patient and hospital factors. Crit Care Explor. (2023) 5(2):e0859. 10.1097/CCE.0000000000000859 36778909 PMC9904767

[13] Hylands M Moller MH Asfar P Toma A Frenette AJ Beaudoin N. A systematic review of vasopressor blood pressure targets in critically ill adults with hypotension. Can J Anaesth. (2017) 64(7):703–15. 10.1007/s12630-017-0877-1 28497426

[14] Tran A Yates J Lau A Lampron J Matar M. Permissive hypotension versus conventional resuscitation strategies in adult trauma patients with hemorrhagic shock: a systematic review and meta-analysis of randomized controlled trials. J Trauma Acute Care Surg. (2018) 84(5):802–8. 10.1097/TA.0000000000001816 29370058

[15] Kudo D Yoshida Y Kushimoto S. Permissive hypotension/hypotensive resuscitation and restricted/controlled resuscitation in patients with severe trauma. J Intensive Care. (2017) 5(1):11. 10.1186/s40560-016-0202-z 34798698 PMC8600688

[16] Chen A Vogan E Foglio J Davis R Reddy AJ Lam SW. Effect of detailed titration instructions on time to hemodynamic stability in ICU patients requiring norepinephrine. Jt Comm J Qual Patient Saf. (2019) 45(9):606–12. 10.1016/j.jcjq.2019.05.003 31320260

[17] Lamontagne F Meade MO Hébert PC Asfar P Lauzier F Seely AJE. Higher versus lower blood pressure targets for vasopressor therapy in shock: a multicentre pilot randomized controlled trial. Intensive Care Med. (2016) 42(4):542–50. 10.1007/s00134-016-4237-3 26891677

[18] Ribeiro AGCD Maitelli AL De M. Valentim RA Leite CRM Soares HB De Almeida NC. Design of an embedded PID controller applied to blood pressure control. Annu Int Conf IEEE Eng Med Biol Soc. (2011) 2011:278–81. 10.1109/IEMBS.2011.6090073 22254303

[19] Urooj S Singh B. Control of mean arterial pressure using fractional PID controller. In: 3rd International Conference on Computing for Sustainable Global Development (INDIACom); New Delhi, India (2016), 1556–9

[20] Hu Y Gullapalli S Abar B Sriram R Patel A Broder J. A Computational Model for Automated Blood Pressure Control in Critical Care. Salt Lake City: American College of Emergency Physicians Scientific Assembly (2025).

[21] Chalumuri YR Sampson CM Shah SA Arabidarrehdor G Tivay A Kinsky M. Comparative assessment of *in vivo* and in silico evaluation of automated fluid resuscitation controllers. Ann Biomed Eng. (2026) 54(3):857–68. 10.1007/s10439-025-03929-2 41355000 PMC12836045

[22] Patel NTP Goenaga-Diaz EJ Lane MR Johnson A Neff M Williams LP. Closed-loop automated critical care as proof-of-concept study for resuscitation in a swine model of ischemia-reperfusion injury. Intensive Care Med Exp. (2022) 10(1):30. 10.1186/s40635-022-00459-2 35799034 PMC9263023

[23] Wikipedia Contributors. Proportional–integral–derivative controller. Wikimedia Foundation. Available online at: https://en.wikipedia.org/wiki/Proportional%E2%80%93integral%E2%80%93derivative_controller# (Accessed August 1, 2026).

[24] Ogata K. Modern Control engineering. 5th ed Boston: Prentice-Hall (2010).

[25] Wikibooks Contributors. Control Systems/System Metrics. (2026). (Available online at: https://en.wikibooks.org/wiki/Control_Systems/System_Metrics (Accessed August 1, 2026).

[26] Banyas P Walker J Gullapalli S Li B Hu Y Bhattacharya S. Autonomous blood pressure regulation for hypertensive crises: outcomes in 10,000 simulated cases. Acad Emerg Med. (2023) 30(S1):258. Available online at: https://onlinelibrary.wiley.com/doi/10.1111/acem.14718

[27] Ziegler JG Nichols NB. Optimum settings for automatic controllers. Trans ASME. (1942) 64:759–68. 10.1115/1.4019264

[28] Aronson S Dyke CM Stierer KA Levy JH Cheung AT Lumb PD. The ECLIPSE trials: comparative studies of clevidipine to nitroglycerin, sodium nitroprusside, and nicardipine for acute hypertension treatment in cardiac surgery patients. Anesth Analg. (2008) 107(4):1110–21. 10.1213/ane.0b013e31818240db 18806012

[29] Sriram R Abar B Patel A Hu Y Gullapalli S Broder J. Automated Hypotensive and Hypertensive Rescue in a Benchtop Fluid Model. Salt Lake City: American College of Emergency Physicians Scientific Assembly (2025).

[30] Smith MD Maani CV. Norepinephrine. Available online at: https://www.ncbi.nlm.nih.gov/books/NBK537259/ (Accessed August 1, 2026).

[31] Aronson S Dasta JF Levy JH Lumb PD Fontes M Wang Y. A cost analysis of the impact of a new intravenous antihypertensive in managing perioperative blood pressure during cardiac surgery. Hosp Pract. (2014) 42(3):26–32. 10.3810/hp.2014.08.1115 25255404

[32] Al-Halawani R Charlton PH Qassem M Kyriacou PA. A review of the effect of skin pigmentation on pulse oximeter accuracy. Physiol Meas. (2023) 44(5):1–22. 10.1088/1361-6579/acd51a PMC10391744 37172609

[33] Ishigami J Charleston J Miller ER 3rd Matsushita K Appel LJ Brady TM. Effects of cuff size on the accuracy of blood pressure readings: the Cuff(SZ) randomized crossover trial. JAMA Intern Med. (2023) 183(10):1061–8. 10.1001/jamainternmed.2023.3264 37548984 PMC10407761

[34] Karges B Rosenbauer J Stahl-Pehe A Flury M Biester T Tauschmann M. Hybrid closed-loop insulin therapy and risk of severe hypoglycaemia and diabetic ketoacidosis in young people (aged 2–20 years) with type 1 diabetes: a population-based study. Lancet Diabetes Endocrinol. (2025) 13(2):88–96. 10.1016/S2213-8587(24)00284-5 39701114

[35] Trappe HJ Gummert J. Current pacemaker and defibrillator therapy. Dtsch Arztebl Int. (2011) 108(21):372–9. quiz 380. 10.3238/arztebl.2011.0372 21691561 PMC3117174

[36] Romagnoli S Ricci Z Quattrone D Tofani L Tujjar O Villa G. Accuracy of invasive arterial pressure monitoring in cardiovascular patients: an observational study. Crit Care. (2014) 18(6):644. 10.1186/s13054-014-0644-4 25433536 PMC4279904

[37] Tsang VM Culwick MD Eley VA. Complications related to arterial line catheters and monitoring reported to webAIRS, 2009–2023. Anaesth Intensive Care. (2026) 54:438–44. 10.1177/0310057X261450643 42290094

[38] Bailey RH Bauer JH Yanos J. Accuracy of disposable blood pressure transducers used in the critical care setting. Crit Care Med. (1995) 23(1):187–92. 10.1097/00003246-199501000-00029 8001371

[39] Kirkendall ES Timmons K Huth H Walsh K Melton K. Human-based errors involving smart infusion pumps: a catalog of error types and prevention strategies. Drug Saf. (2020) 43(11):1073–87. 10.1007/s40264-020-00986-5 32797355 PMC7750013

[40] Schnock KO Dykes PC Albert J Ariosto D Call R Cameron C. The frequency of intravenous medication administration errors related to smart infusion pumps: a multihospital observational study. BMJ Qual Saf. (2017) 26(2):131–40. 10.1136/bmjqs-2015-004465 26908900

[41] Ohashi K Dalleur O Dykes PC Bates DW. Benefits and risks of using smart pumps to reduce medication error rates: a systematic review. Drug Saf. (2014) 37(12):1011–20. 10.1007/s40264-014-0232-1 25294653

[42] Silva MS Araújo JL Nunes GAMA Rosa MFF Luz GVS Rosa SSRF. Precision and reliability study of hospital infusion pumps: a systematic review. Biomed Eng Online. (2023) 22(1):26. 10.1186/s12938-023-01088-w 36932393 PMC10023007

[43] Son H-J Kim S-H Ryu J-O Kang M-R Kim M-H Suh J-H. Device-related error in patient-controlled analgesia: analysis of 82,698 patients in a tertiary hospital. Anesth Analg. (2019) 129(3):720–5. 10.1213/ANE.0000000000003397 31425211

[44] Digital Health Terms. U.S. Food and Drug Administration. (2022). Available online at: https://www.fda.gov/medical-devices/digital-health-center-excellence/digital-health-terms (Accessed September 9, 2026).

[45] Special 510(k): Device Modification Inspiration>” Ventilator System. (2008). Available online at: https://www.accessdata.fda.gov/cdrh_docs/pdf7/K072590.pdf (Accessed September 9, 2026).

[46] Zhang J Johnson TR Patel VL Paige DL Kubose T. Using usability heuristics to evaluate patient safety of medical devices. J Biomed Inform. (2003) 36(1-2):23–30. 10.1016/s1532-0464(03)00060-1 14552844

[47] Kusano KD Scanlon JM Chen Y-H McMurry TL Chen R Gode T. Comparison of Waymo rider-only crash data to human benchmarks at 7.1 million miles. Traffic Inj Prev. (2024) 25(sup1):S66–77. 10.1080/15389588.2024.2380786 39485678

[48] Williams PA Woodward AJ. Cybersecurity vulnerabilities in medical devices: a complex environment and multifaceted problem. Med Devices (Auckl). (2015) 8:305–16. 10.2147/MDER.S50048 26229513 PMC4516335

[49] Axelrod R Iliev R. Timing of cyber conflict. Proc Natl Acad Sci U S A. (2014) 111(4):1298–303. 10.1073/pnas.1322638111 24474752 PMC3910638

[50] General Principles of Software Validation. U.S. Food & Drug Administration. Available online at: https://www.fda.gov/regulatory-information/search-fda-guidance-documents/content-premarket-submissions-device-software-functions (Accessed September 9, 2026).

[51] Quality Management System Regulation (QMSR). U.S. Food & Drug Administration. Available online at: https://www.fda.gov/medical-devices/postmarket-requirements-devices/quality-management-system-regulation-qmsr (Accessed September 9, 2026).

